# Clinical Applications of *Polypodium leucotomos* (Fernblock^®^): An Update

**DOI:** 10.3390/life13071513

**Published:** 2023-07-05

**Authors:** Azahara Rodríguez-Luna, Alicia Zamarrón, Ángeles Juarranz, Salvador González

**Affiliations:** 1 Department of Basic Health Sciences, Faculty of Health Sciences, Universidad Rey Juan Carlos (URJC), 28933 Alcorcón, Spain; 2 Department of Pharmacology, Faculty of Pharmacy, University of Seville, 41012 Seville, Spain; 3 Department of Biology, Faculty of Sciences, Autónoma University of Madrid (UAM), 28049 Madrid, Spain; aliszm@gmail.com (A.Z.); angeles.juarranz@uam.es (Á.J.); 4 Department of Medicine and Medical Specialties, Alcalá de Henares University, 28805 Madrid, Spain; salvagonrod@gmail.com

**Keywords:** *Polypodium leucotomos*, Fernblock^®^, photoprotection, photodermatoses, photoaging, hyperpigmentation

## Abstract

Exposure to sun radiation leads to higher risk of sunburn, pigmentation, immunosuppression, photoaging and skin cancer. In addition to ultraviolet radiation (UVR), recent research indicates that infrared radiation (IR) and visible light (VIS) can play an important role in the pathogenesis of some of these processes. Detrimental effects associated with sun exposure are well known, but new studies have shown that DNA damage continues to occur long after exposure to solar radiation has ended. Regarding photoprotection strategies, natural substances are emerging for topical and oral photoprotection. In this sense, Fernblock^®^, a standardized aqueous extract of the fern *Polypodium Leucotomos* (PLE), has been widely administered both topically and orally with a strong safety profile. Thus, this extract has been used extensively in clinical practice, including as a complement to photodynamic therapy (PDT) for treating actinic keratoses (AKs) and field cancerization. It has also been used to treat skin diseases such as photodermatoses, photoaggravated inflammatory conditions and pigmentary disorders. This review examines the most recent developments in the clinical application of Fernblock^®^ and assesses how newly investigated action mechanisms may influence its clinical use.

## 1. Introduction

Photoprotection is the first-line prevention strategy to avoid the development of skin cancer and premature aging. Ultraviolet radiation (UVR) promotes skin cancer by inducing DNA damage, triggering inflammatory processes and causing immunosuppression and plays part in premature aging through alterations in extracellular matrix network and remodeling. Most of these detrimental effects are mainly mediated by generation of reactive oxygen species (ROS) and the consequent oxidative stress. Adoption of specific behaviors (such as wearing protective clothes, hats and sunglasses, and avoiding excessive sun exposure) and the use of topical sunscreens are the most common measures to counteract the harmful effects of UVR. Although traditional sunscreens are a critical component in all photoprotective regimens, they have limitations (inadequate application and need for frequent reapplication, short half-life, lack of photostability and insufficient protection against all wavelengths, among others) and have also increasingly been questioned for their safety and their impact on the environment [1,2]. In this sense, photoprotection can be provided not only by topical sunscreens but also by oral administration of substances (such as polyphenols, carotenoids and other antioxidants) that are being identified as systemic photoprotection agents in humans [2]. A well-known photoprotective agent is the standardized aqueous extract from the leaves of the fern *Polypodium leucotomos* (PLE or Fernblock^®^ (trademark name)). *Polypodium leucotomos* (PL) is a fern of the Polypodiaceae family, genus Phlebodium, native to Central and South America, where it has had a historical role in traditional medicine, especially for the treatment of skin diseases. A standardized aqueous extract from the leaves of the fern PL (PLE), rich in polyphenols and specifically in phenolic acids, has been developed to exploit the photoprotective properties of the plant and to provide a steady phenolic content. This extract was introduced as Fernblock^®^ in Europe in the year 2000, both in topical and oral forms, and is currently available in more than 26 countries, including the U.S., as a dietary supplement, since 2006.

**Figure 1:**
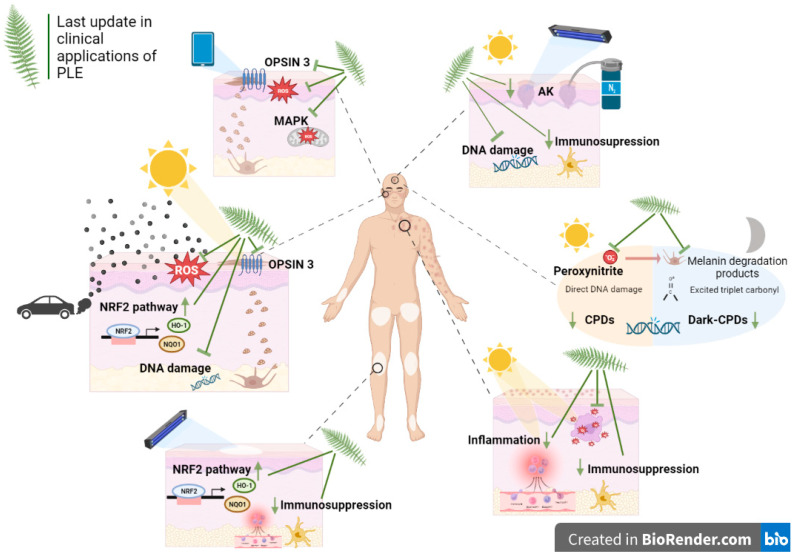
The graphical summary highlights the latest relevant studies concerning Fernblock^®^, represented by the image of the fern, and examines their implications for its clinical application. The image includes abbreviations such as MAPK (mitogen-activated protein kinase), AK (actinic keratosis), NRF2 (nuclear factor erythroid-2-related factor 2), and CPDs (cyclobutane pyrimidine dimers), which correspond to specific molecular pathways or biomarkers related to Fernblock^®^ and its effects. Created with BioRender.com.

Phenolic compounds identified in PLE are 4-hydroxybenzoic acid, 3,4-dihydroxybenzoic acid (protocatechuic acid), 4-hydroxy-3-methoxybenzoic acid (vanillic acid), 3,4-dihydroxycinnamic acid (caffeic acid), 4-hydroxycinnamic acid (p-coumaric),3-methoxy-4-hydroxycinnamic acid (ferulic acid), 4-hydroxycinnamoylquinic acid and five chlorogenic acid isomers [3]. Of these, ferulic and caffeic acids are the most potent antioxidants. However, it is important to note that González et al. demonstrated in 2018 that there were significant differences between different PL extracts which could be attributed to the specific plant part used, the method of extraction and the plant’s origin and growth conditions. Generally, extracts from the leaves are more potent and yield more meaningful outcomes. Nonetheless, this research also suggested that other moieties, whether antioxidant or not, may have a critical role in the function of these extracts as dietary supplements with antiaging and antioxidant properties [4,5]. As mentioned earlier, this extract is rich in polyphenols and specifically in phenolic acids and has been developed to exploit the photoprotective properties of the PL fern and to provide a steady phenolic content. Its solid mechanism of action, its success in clinical trials, and the increased social interest in natural substances, such as polyphenols, have placed PLE as an interesting photoprotective, antioxidant and anti-inflammatory option (Figure 1). In this regard, numerous studies have been carried out to prove the role of the aqueous PLE in photoprotection, which have been summarized by many authors [6,7].

The aim of this review is to establish the current state of the art regarding the uses of PLE and provide an interpretive synthesis that describes how recent advances may influence its clinical applications. In certain cases, referencing older studies considered fundamental in the study area can also provide valuable insights and improve our understanding of newly obtained findings. Additionally, we aim to determine the potential future directions of PLE research, with special emphasis on the role of Fernblock^®^ as an adjuvant in photoprotection. Finally, we will explore the newly researched action mechanisms that may impact its clinical management.

## 2. Materials and Methods

This state-of-the-art narrative review was developed in accordance with the guidelines published by Barry et al. (2022) [8]. The first step in our search process was to build an initial pool of articles, for which we searched Pubmed, Scopus and Google Scholar databases, though we prioritized Pubmed Central to be the National Library of Medicine of reference. The main criterion to select articles was the inclusion of PLE, using the following keywords: *Polypodium leucotomos*, Fernblock^®^, photoprotection, photodermatosis, skin pigmentation and skin cancer. In all searches, keyword combinations included either the term “*Polypodium leucotomos*” or the term “Fernblock^®^”. We limited our search to articles published between 2019 and 2023 and written in English. From the 224 collected articles, 53 were excluded due to various reasons, such as duplication, erratum, being outside the scope, or unavailability of full-text copies. Among the 171 selected articles that met the specified criteria, 34 were eliminated due to their brief mention of PLE without providing significant relevance. The remaining articles, totaling 120, were included in the tables, while 17 articles are cited throughout the text as they contained more comprehensive information regarding PLE.

## 3. Results and Discussion

### 3.1. PLE Photoprotective Activity

Numerous compounds have been demonstrated to have a protective effect against various harmful effects of UV radiation, including photocarcinogenesis, sunburn, photoaging, and UVB-mediated phototoxicity. These effects result from the modulation of different pathways as demonstrated in different in vitro and in vivo models [9]. Briefly, we can affirm that PLE’s activity has been widely studied, and it has been attributed to several action mechanisms. PLE improves the skin’s endogenous antioxidant system by reducing lipid peroxides and neutralizing ROS and free radicals, especially superoxide anions and hydroxyl radicals generated after exposure to UV and VIS radiation, unlike traditional antioxidants such as vitamin C, E, or carotenoids, which are mainly effective against singlet oxygen. Moreover, Fernblock^®^ increases the activity of the nuclear factor erythroid-2-related factor 2 (NRF2) transcription factor and its associated antioxidant targets, which is linked to its capacity to decrease inflammation, melanin production, and overall cell damage [10].

In the context of UVR-induced inflammation, the basis of its anti-inflammatory properties is its ability to inhibit the expression of the tumor necrosis factor α (TNF-α), iNOS, redox-sensitive transcriptional factors, activator protein 1 (AP-1) and nuclear factor kB (NF-kB) [11]. PLE also decreases the expression of COX-2 and PGE2 [12]. Over the years, many indicators have been examined which provide essential information for verifying the photoprotective effect that has been demonstrated through clinical and preclinical studies. However, the effect of PLE on AP-1 and NF-kB expression after exposure to solar simulated radiation (SSR) cannot be explained only by the antioxidant action of PLE since treatment with a bona fide antioxidant does not decrease AP-1 and NF-kB expression in human keratinocytes subjected to SSR [11].

### 3.2. Clinical Applications

The successful results of Fernblock^®^ in clinical trials, together with its action mechanisms and growing interest in natural substances like polyphenols, have positioned PLE as a promising option for photoprotection, antioxidant and anti-inflammatory treatment and as an adjuvant therapy for various pathologies [2,6]. In the upcoming sections, we will examine the research progress supporting its application in these conditions, and we will suggest a future scenario based on evidence.

#### 3.2.1. Oncodermatology

In the field of oncology, a recent review by Calzari et al. (2023) elucidates the ways in which PLE functions and assesses its applications in oncodermatology, with reference to both in vitro and in vivo research [13]. However, this review is not the only one, as the trend in recent years has been to publish literature reviews demonstrating the photoprotective effects of PLE, thereby confirming its therapeutic potential against various types of cancerous growths. Alongside these reviews, there have been seven experimental studies, four of which were clinical and three preclinical, which can be found summarized in Table 1.

Accordingly, a wide range of earlier studies are recognized as crucial references that have significantly advanced our understanding of the potential of Fernblock^®^ in the field of cancer prevention in both mice and humans. PLE inhibited UVR-mediated DNA damage and mutagenesis through a double mechanism that consisted of prevention of cyclobutane pyrimidine dimer (CPDs) accumulation and reduction of 8-OH-dG and H2Ax, thus preventing oxidative damage [4]. Also, PLE decreased UVA-dependent mitochondrial DNA damage by reducing common deletions (CD) [14]. In vitro and in vivo studies suggest that PLE may have a role in the treatment of UV-induced skin inflammation and cancer, probably due to its antioxidant and p53-activating properties [15]. It is important to note that the extracellular matrix (ECM) provides structural integrity to the tissue and is remodeled during skin aging/photoaging and cancer. In vitro experiments showed that PLE directly inhibited the enzymatic activity and expression of MMPs in melanoma cells and fibroblasts and stimulated the expression of tissue inhibitors of metalloproteinases (TIMPs) in melanoma cells, reducing melanoma cell growth and ECM remodeling [16,17]. Also, clinical studies shown that PLE reduces epidermal cell proliferation and the number of cyclin D1- and PCNA-positive epidermal cells caused by UVR exposure [18,19]. In relation to the process of cancerization, previous research has shown that taking oral PLE supplements following PDT can improve AK clearance and reduce recurrence as compared to PDT alone [20].

However, three recent studies have been conducted to determine the effectiveness of PLE in the context of cancerization not only by oral administration but also by topical administration. The first of them investigated the effectiveness of a new medical device (NMD) in treating the field cancerization in 30 patients with Aks after PDT. The NMD contained a complex of DNA-repair enzymes, UV-filters and Fernblock^®^, while the control group received a standard sunscreen (SS). The study utilized clinical, dermoscopic, reflectance confocal microscopy (RCM) and histological evaluations to assess the outcomes and found that after six and twelve months of treatment, the SS group showed a significant increase in the number of AKs compared to the NMD group. The NMD group also showed a significant reduction in the extension and grade of atypia compared to the SS group. Histopathological evaluation showed an improvement in keratinocyte atypia grade in all groups after six months of PDT, but p53 expression was significantly lower in the NMD group at twelve months compared to the SS group. Overall, the NMD was well-tolerated with no serious adverse events reported [21]. Another recent prospective clinical study evaluated the effectiveness of the same formulation in individuals with AK who underwent cryotherapy. The evaluation involved measuring changes in the AKASI score (Actinic Keratosis Area and Severity Index) and utilized non-invasive line-field confocal-optical coherence tomography (LC-OCT) analysis. The findings revealed that the use of the sunscreen containing DNA-repairing enzymes and PLE significantly reduced the AKASI score after 3 and 12 months treatment compared to the control group. Consequently, the study concluded that the PLE-based sunscreen considerably improved AKASI score among individuals receiving cryotherapy treatment [22]. Finally, a recent prospective, multicenter, randomized controlled trial was conducted to compare not only the effectiveness of a sunscreen with Fernblock^®^ vs. one without but also the impact of oral photoprotection for managing AKs in elderly individuals with severe actinic damage. The group that received both topical sunscreen with Fernblock^®^ and Fernblock^®^ oral supplementation showed the most significant improvements in AK and field cancerization parameters compared to control group (which used a standard topical sunscreen). These results suggest that combining oral and topical photoprotection leads to superior clinical and anatomical outcomes [23]. In summary, these studies provide evidence to suggest that both oral and topical PLE could be utilized as an adjuvant treatment option for field cancerization. However, it is necessary to conduct further research in order to validate its effectiveness when compared to established and widely accepted medications considered to be the gold standard.

Regarding melanoma, Aguilera et al. (2013) also investigated the protective role of oral administration of PLE in patients at risk of malignant melanoma (MM) and in the interaction between MC1R polymorphisms and the cyclin-dependent kinase (CDK) inhibitor 2A gene (CDKN2A) status with MED 25–50%. Among patients with familial MM, those individuals with mutations in CDKN2A and/or MC1R had greater differences regarding the response to treatment with PLE [24]. According to these results, the authors indicated that patients with higher UVR sensitivity (lower basal MED) would benefit the most from oral PLE treatment. These results are intriguing, and thus studies with long-term PLE administration in patients with a high risk of developing MM would be important to expand and confirm these data. No recent clinical studies have been reported in this field that confirm the effectiveness of PLE in preventing melanoma. However, there are new pre-clinical studies which help uncover new mechanisms of action in relation to this matter (Table 1). Within the scope of the latest in vitro studies, three principal works have been incorporated to the scientific approach. The first one explores the potential of a dietary supplement containing sulforaphane (SFN) and Fernblock^®^ extract, in terms of its antioxidant, antineoplastic and antiaging properties. The study analyzed the impact of SFN/FB combination on MMPs, ROS production, and IL-1β secretion in human normal keratinocytes. The combination of these actives was found to be more effective than each on its own in inhibiting melanoma cell migration in vitro, MMP-1, -2, -3 and -9 production, inflammasome activation and IL-1β secretion. Moreover, when used in normal keratinocytes with a pro-inflammatory stimulus like TNF-α, SFN/FB was more efficient in inhibiting MMP-1 and -3 production and IL-1β secretion than SFN or FB alone. Based on these results, the authors suggested that SFN/FB-based supplements could be used as potential preventive measures against skin aging and as adjuvants in the treatment of advanced melanoma [25].

The second work represents an important step forward with respect to understanding the mechanisms involved in DNA damage, and in particular the formation of dark CPDs. Initially discovered by Premi el al (2015), this recent work performed by Portillo-Esnaola et al. (2021) confirms that UVA radiation triggers DNA damage in melanocytes even hours after sun exposure has ended due to increased production of nitrogen reactive species (NO•, O2− and ONOO−), which is linked with the increased formation of CPDs and dark-CPDs. UVA-induced significant dark-CPD formation was observed as soon as 3 h after exposure and the highest peak of dark-CPD formation was obtained 24 h after exposure. However, pre-treatment with Fernblock^®^ (0.3–0.75 mg/mL) was found to reduce the production of these reactive species and the formation of dark-CPDs due to its antioxidant and scavenging properties. We now understand that PLE not only prevents sunlight-induced DNA damage but also offers protection against it even after exposure to solar irradiation. This suggests that Fernblock^®^ could be a promising candidate to complement traditional sunscreens in providing long-lasting skin protection against dark-CPD formation formed after irradiation [26,27].

The third study, performed by Gallego-Rentero et al. (2022) is related to DNA damage induced by photopollution. The interaction of UVA radiation with environmental pollutants, specifically those of a polycyclic aromatic hydrocarbon (PAH) nature such as benzo[a]pyrene (BaP), produces what is known as photopollution. BaP acts as a photosensitizer and upon absorption of UVA radiation it causes increased cell damage in vitro and tumorigenicity in mice even at non-toxic concentrations. Thus, the study evaluated the protective effect of Fernblock^®^ against the combination of pollution and UVA radiation in human keratinocyte and mouse melanocyte cell lines. This preclinical study demonstrated the efficacy of Fernblock^®^ in preventing changes in cellular structure, viability, oxidative stress, and DNA damage. These findings provide strong evidence that Fernblock^®^ induces the priming of cells, rapidly promoting the activation of repair mechanisms and efficient elimination of oxidized derivatives that appear in the nuclear DNA as a result of sequential exposure to BaP and UVA light [28].

In order to enhance photoprotection, it is crucial to explore innovative methods that move beyond conventional measurements of minimal erythema dose (MED). The first evaluation focuses on demonstrating the clinical impact of assessing the immunomodulatory and preventive effects of DNA damage through in vivo studies. Thus, Schalka and Donato (2019) clinically evaluated the efficacy of an SPF 90 sunscreen with PLE in protecting against sun-induced skin damage vs. the same formulation without PLE. The presence of PLE provided additional protection, further reducing erythema, pigmentation, DNA damage, collagen breakdown and immunosuppression vs. placebo [29]. One of the most significant findings from this study is the marked reduction of p53 in skin areas protected with SPF 90 sunscreen containing Fernblock^®^ indicating reduced DNA alteration. These findings were completed by Aguilera et al. (2021), who conducted an in vitro study that analyzed the impact of Fernblock^®^ as a part of topical sunscreen in protecting the skin from photoimmunosuppression and other detrimental biological effects caused by exposure to UV radiation. In addition to the biological activity demonstrated in previous studies, the UV absorption properties of PLE provide an additional booster effect to topical sunscreens, increasing SPF and UVAPF and enhancing protection against not only erythema and permanent pigment darkening reaction but also against immunosuppression [30].

Regarding xeroderma pigmentosum (XP), data has shown that PLE reduces UVR-induced COX-2 levels, at least in part through activation of p53, and decreased epidermal cell proliferation induced by UVR in a mouse model [15]. A case report on XP treatment demonstrated the efficacy of a topical film-forming medical device containing a DNA-repair enzyme, photolyase (Repairsomes^®^) and very high protection UV filters in preventing the growth of skin cancer lesions in patients with XP [31].

Finally, it is worth highlighting the recent findings of Lacerda et al. (2023) in the field of oral cancer prevention. Their study demonstrated that PLE has the ability to suppress oral cancer cell growth in vitro in SCC-9, SCC-15 and SCC-25 cell lines and prevent tumor development in vivo in mice with induced oral carcinogenesis. A decrease in the expression of Ki67 and PCNA proliferating markers as well as in N-cad (Cdh2), Vim and Twist markers related to migration was observed in tongue tissues. Therefore, PLE may have a beneficial effect on immune and inflammatory responses related to oral tumors and could be a promising natural therapeutic approach for preventing and treating oral cancer due its immunomodulatory activity [32].

**Table 1:** This table provides a summary of the scientific articles published in the last five years in the field of oncodermatology, specifically highlighting the references to, and conclusions about, PLE treatment.

Oncodermatology			
Design	Pathology/Focus	Summary/Outcome	Study Reference
Review	General oncodermatology	This review reports the mechanisms through which *Polypodium leucotomos* acts to evaluate its uses in oncodermatology with references to in vitro and in vivo studies.	[13]
Review and book chapter	Continuing medical education about skin cancer and sunscreen use	These reviews provide evidence-based recommendations for the use of sunscreen as a preventive strategy against skin cancer while also considering potential risks and environmental impacts associated with the use of some chemical sunscreen filters. PLE is included as a reference oral sunscreen technology for prevention of photodamage.	[33,34,35,36,37]
Reviews and book chapter	Botanical interventions for photoprotection and skin cancer	These works review the main actives derived from plants with scientific evidence as treatment in photoprotection and offer an overview of cancer and phytotherapy. Specifically, they review the existing literature on the properties of PLE and its potential therapeutic effects in preventing skin damage. These reviews include studies conducted in vitro, in vivo and clinical trials.	[38,39,40,41,42]
Review	Preventive interventions for keratinocyte carcinoma	This manuscript examines the potential of pharmaceuticals, plant-derived phytochemicals and vitamins for preventing keratinocyte carcinoma. One such reference photoprotectant is PLE, which has been shown to inhibit the development of tumors and acute UV-induced damage in humans.	[43]
Clinical study	Field cancerization	This clinical study suggests that a new medical device treatment containing Fernblock^®^ (NMD) is a useful treatment method for improving the precancerous field and preventing the development of new AKs.	[21]
Clinical study	Oral cancer	The findings indicate that PLE has the ability to suppress oral cancer cell growth in vitro and prevent tumor development in vivo. Thus, PLE could be a promising natural therapeutic approach for preventing and treating oral cancer.	[32]
Preclinical study	Skin cancer markers	This in vitro study suggests that FB could be a promising candidate to complement traditional sunscreens in providing long-lasting skin protection against dark-CPDs formation after irradiation.	[26]
Preclinical study	Melanoma	This in vitro research suggests that supplements containing sulforaphane/FB could be used to prevent skin aging and help treat advanced melanoma.	[25]
Preclinical study	Skin cancer induced by photopollution	This preclinical study demonstrates the efficacy of PLE in preventing changes in cellular structure, viability, oxidative stress and activation of the melanogenic signaling pathway caused by exposure to both BaP and UVA light.	[28]
Actinic keratosis			
Review	Actinic keratosis	This review article examines in vitro experiments and clinical trials that utilize evidence-based therapeutic methods before or after photodynamic therapy (PDT). Specifically, the effectiveness of topical treatments and oral supplementation, such as diclofenac, imiquimod and PLE, among others, as well as mechanical-physical treatments, are evaluated.	[44]
Review	Actinic keratosis	In this article, the authors offer expert opinions and practical insights into the treatment of actinic keratosis and field cancerization using monotherapy or a combination of therapies among which PLE is cited. The primary objective is to achieve improved, quicker and more tolerable clinical outcomes.	[45]
Review	Actinic keratosis	This review discusses various physical ablative techniques and drug preparations available for treatment. It emphasizes the need for careful evaluation of efficacy, toxicity and tolerability data, as well as practical considerations such as treatment protocols and patient preferences, to achieve maximal adherence and prevent treatment failure. It includes PLE as a chemopreventive treatment tool against the development of AK.	[46]
Xeroderma pigmentosum			
Review	Xeroderma pigmentosum	The purpose of this review is to present the symptoms, diagnosis, and treatment of XP. It also includes oral PLE as a treatment adjuvant due to its chemoprotective, antioxidative, anti-inflammatory and immunomodulatory properties. All these effects have the potential to lessen the phototoxic effects of UVR and thus reduce UVR-induced skin damage and cancer.	[47]

#### 3.2.2. Photodermatoses and Photoaggravated Skin Diseases

Photosensitivity occurs when there is an abnormal reaction between a specific component of the sun’s electromagnetic spectrum such as UVR (UVA, UVB) or VIS, and chromophores in the skin. UVA is the most common type of sunlight that leads to photosensitivity, while exposure to VIS may trigger a condition called porphyria. The causes of photodermatosis can be varied: some types are caused by autoimmune reactions, while others are triggered by drugs or connective tissue disease. Additionally, certain types of photodermatosis can be caused by abnormal inherited biochemical pathways [48]. It is widely recognized that exposure to UV radiation can cause changes to both the skin and overall immune system. Regulatory T (Treg) cells play a crucial role in maintaining immune homeostasis by suppressing immune responses to both self- and non-self antigens [49].

Regarding photo-immunosuppression, a multitude of older studies represent essential references that have greatly enhanced our comprehension of the potential of PLE in treating photodermatoses. PLE is endowed with immunomodulatory properties acting as a photoimmunoprotective agent through different mechanisms. PLE prevents UCA isomerization from trans to cis isomer which is a triggering event of skin immunosuppression [50]. PLE also prevents epidermal Langerhans cells (eLC) depletion caused by UV irradiation in vivo [51]. Multiple molecular mechanisms may underlie the increase in survival of dendritic cells, including inhibition of UCA isomerization as mentioned above, inhibition of iNOS expression [11] and improvement of endogenous systemic antioxidant systems [52]. Consequently, PLE is able to reduce the infiltration of neutrophils and macrophages [12] and decrease inflammatory molecules in humans [19] and mice [51], thus inhibiting mast cells and leukocyte extravasation in the irradiated area. The immunomodulation of these markers has a significant clinical impact because reducing the infiltration of neutrophils and macrophages can directly affect the inflammatory response of the skin to UV or visible light radiation. This exaggerated response is observed in immunologically mediated photodermatoses (previously referred to as idiopathic), and controlling the release of these markers can directly impact these skin disorders, as they are aggravated by greater inflammatory responses.

Concerning photodermatoses and photoaggravated diseases, there is a need to increase clinical research on various conditions such as lupus erythematosus, polymorphous light eruption (PMLE), rosacea, solar urticaria, different forms of dermatitis, and psoriasis, among others. Given the known action mechanisms of PLE and its demonstrated beneficial effects in the prevention of photodermatoses, expanding the existing clinical evidence on the impact of PLE in these pathologies would be worthwhile, especially considering their high prevalence [53]. Another important area to continue research is photodermatosis induced by chemical agents, as it is one of the primary issues faced by society. There are over 300 drugs classified as phototoxic, which undergo a photochemical reaction when the skin is exposed to radiation and become chemically excited. This leads to a reaction with other molecules in the skin environment, such as free radicals, proteins and enzymes, causing phototoxicity and cell damage. This can result in photodermatosis and may also lead to DNA damage and skin cancer. Moreover, these drugs can generate inflammatory reactions known as photoallergy. Phototoxic drugs are widely used and are taken by a significant proportion of the population. Among the phototoxic drugs are antihypertensive drugs, antidiabetic drugs, NSAIDs, antibacterial drugs and others [54]. On light of all this, this review highlights the potential clinical effects of PLE on various types of photodermatosis, providing a summary table with the most recent studies conducted on the topic (Table 2).

##### Polymorphous Light Eruption and Actinic Prurigo

Polymorphous light eruption (PMLE) is a frequently occurring skin condition caused by an immune response triggered by exposure to UVR from sunlight, which can lead to a range of alterations due to the breakdown in the body’s ability to suppress the immune response. It has been found that the combination of Fernblock^®^, nicotinamide, vitamin D and zinc can reduce the intensity of pruritus and the severity of flare-ups in 87% of PMLE patients [55].

Although less common than other photodermatoses, actinic prurigo is also characterized as a condition where exposure to sunlight triggers an immune response. As in the case of PMLE, the treatment of actinic prurigo with PLE leads to a significant improvement in this disease: duration and severity of skin eruptions are reduced [56]. Recent research suggests that Th1 cells and TNF-α are significant contributors to the development of actinic prurigo. As a result, PLE may have a vital role in reducing actinic prurigo by regulating the immune response [57].

##### Solar Urticaria and Photosensitive Lupus

Solar urticaria (SU) is a rare chronic acquired photosensitivity disorder that causes recurrent episodes of urticaria rash on skin areas exposed to sunlight. Although usually a benign condition, it may be extremely disabling and limiting for patients. Its pathogenesis has not yet been adequately understood and it can be difficult to diagnose. In 2019, Photiou et al. performed a retrospective review of 83 patients identified as having SU. Of the total number of patients who underwent the monochromator test, SU was confirmed in 58%, and most of them reacted to VIS and UVA or UVA alone. Among the treatment strategies for SU, antihistamines and sun avoidance are the most prescribed. In difficult-to-treat SU patients who do not respond to these strategies, other options such as the monoclonal anti-immunoglobulin E antibody omalizumab have been shown to be effective. Also, PLE is suggested as a safe treatment option in SU [58].

The findings of this study are consistent with prior research conducted by Caccialanza et al., reflected in two clinical studies in 2007 and 2011. These studies assessed the effectiveness of orally administered PLE in the treatment of SU. The first study involved two patients diagnosed with SU: the intake of PLE at a dose of 480 mg/day significantly reduced the skin reaction to sunlight and improved the related symptoms such as prurito [59,60]. In the second study, the four SU patients enrolled were also treated with 480 mg/day of oral PLE and the same benefits were observed, without side effects or tolerance problems [59,60]. All this makes PLE an effective and safe treatment for photoprotection in this idiophatic photodermatosis [59,60].

Photosensitive lupus is another form of photo-exacerbated dermatosis. It consists of a multifactorial inflammatory and autoimmune disease with a variety of clinical manifestations of differing severity. In 2022, Malara et al. conducted a review of the effects of the anti-inflammatory drug thalidomide in patients with discoid lupus erythematosus (DLE) who were refractory to different medications. In two of the patients included in this study who present painful erythematous lesions, 50 mg daily of thalidomide was administered along with PLE capsules and sunscreen with remarkable clinical results. Patients who received PLE experienced a longer-lasting clearance of symptoms. As incorporating this natural active helped to reduce the side effects associated with thalidomide treatment, PLE could serve as a safety measure to lower thalidomide dosage [61].

Additionally, Segars et al. hypothesized the use of PLE as an immunomodulatory agent to adjunct the treatment of subacute cutaneous lupus erythematosus (SCLE), another type of photosensitive lupus [62]. Previous data from a case report by Breithaupt et al. (2012) presented new evidence of a beneficial clinical effect of PLE in a patient with moderately controlled subacute cutaneous lupus erythematosus on hydroxychloroquine. This patient achieved near total remission with the addition of PLE to her treatment regimen, suggesting it might have future applications in photosensitizing dermatoses, including other forms of cutaneous lupus erythematosus [63].

The clinical findings of these studies align with the mechanisms discussed in a recent review by McCarty et al., indicating that PLE, with its high content of phenolic compounds and antioxidant activity, has the potential to suppress superoxide anions, lipoperoxides, and hydroxyl radicals. Additionally, PLE shows promise as an anti-inflammatory agent and an immune modulator with therapeutic applications [9].

**Table 2:** This table provides a summary of the scientific articles published in the last five years in the field of photodermatosis, specifically including references to, and conclusions about, PLE treatment.

Photodermatoses and Photoaggravated Skin Diseases			
Design	Pathology/Focus	Summary/Outcome	Study Reference
Review	UVB phototoxicity	The objective of the text is to explore and discuss the potential of various nutraceuticals in preventing or mitigating the effects of phototoxicity caused by UVB radiation. The text provides the mechanisms by which these nutraceuticals, including spirulina, soy isoflavones and PLE, among others, may offer protection against UVB-induced sunburn, photoaging and NMSC.	[9]
Review	Photodermatoses	This chapter outlines various topical and systemic agents that can trigger phototoxic and photoallergic reactions. In terms of treatment, the chapter mentions that PLE can be used as a systemic antioxidant in conjunction with PUVA to manage cases of PMLE.	[64]
Review	Idiopathic photodermatoses	This chapter provides a clinical approach to managing idiopathic photodermatoses, including conditions such as PMLE, actinic prurigo and idiopathic solar urticaria, among others. Preventing and managing these conditions involves implementing photoprotective measures and increasing the skin’s tolerance to sunlight through the use of narrow-band UVB therapy and other forms of phototherapy or photochemotherapy when necessary. In addition, topical or systemic antioxidants like PLE may be helpful in certain cases.	[65]
Review	Diet and Photodermatoses	Prior studies have explored the connection between diet and several skin conditions, including rosacea, hidradenitis suppurativa, herpes labialis and vitiligo. The authors consolidate the findings from existing literature to create clear and concise guidance regarding dietary supplements that could be beneficial or harmful. By doing so, they provide healthcare professionals with evidence-based recommendations to assist their patients, including PLE as a recommended supplement in the treatment of vitiligo.	[66]
Case report	Photodermatoses	The case study involves a 55-year-old man who experienced a severe and painful skin eruption with erythema and blisters in sun-exposed areas one month after starting vandetanib treatment. Despite treatment with steroids and avoiding sun exposure, the condition did not improve until the patient began taking oral supplements of PLE. This case highlights the potential of PLE as a safe and effective photoprotective agent for treating refractory phototoxic reactions.	[67]
Polymorphous light eruption and actinic prurigo			
Review	PMLE	The purpose of the review is to provide a better understanding of the molecular pathogenesis of PMLE by examining the immunological disturbances associated with the disease. The authors emphasize the potential of PLE as an immunomodulatory and antioxidant agent and suggest it could be used as a preventive therapeutic approach for PMLE treatment.	[53]
Review	PMLE	The goal of this article is to provide readers with the latest information on PMLE with regards to its epidemiology, clinical presentation, underlying pathophysiology, available treatments and prognosis. PLE is presented as a potential treatment for PMLE, and the review cites open-label studies showing that this supplement can reduce the severity, frequency and rapidity of onset of PMLE reactions.	[68]
Review	Actinic prurigo	The aim of this study is to provide a summary of current knowledge related to two types of photodermatoses—actinic prurigo (AP) and hydroa vacciniforme (HV), both of which typically develop during childhood. Among suggested treatment, botanical agents such as PLE may be beneficial in reducing photosensitivity in certain skin conditions like PMLE and solar urticaria. However, further studies are needed to suggest their usefulness in treating AP.	[57]
Case report	Actinic prurigo	In this report, the authors describe the successful use of PLE in an 11-year-old girl with AP. PLE treatment led to a significant reduction in her symptoms and no negative side effects were observed. PLE has a wide-ranging impact on the immune system and acts as an antioxidant by promoting an anti-inflammatory environment.	[56]
Solar Urticaria and Photosensitive Lupus			
Restrospective analysis	Solar urticaria (SU)	The authors conducted a retrospective analysis in 83 patients with SU. Among the 60 patients who underwent monochromator testing, 35 were confirmed to have SU, with most reacting to VIS and UVA, or UVA alone. The mainstay of treatment for SU is antihistamines and sun avoidance. However, for patients who do not respond to these treatments, other options such as omalizumab may be of potential interest. Also, PLE is sugested as a treatment option for SU, without side effects.	[58]
Review	Lupus	This review analyses natural actives traditionally used to treat rheumatological conditions, including antimalarials, which could also be beneficially indicated for cutaneous lupus eryhtematosus (CLE). It also suggests their combination with PLE as a photoprotective supplement to control photosensitivity.	[69]
Review	Lupus	This text reviews the available evidence regarding local and systemic therapies for CLE and provides healthcare professionals with alternative treatment options for patients who were previously treated with quinacrine, which is currently unavailable in the USA. Among these options, PLE is proposed with a level of evidence of 5 in accordance to the levels adapted from the Oxford Centre for Evidence-Based Medicine.	[70]
Clinical cases	Lupus	This article examines the use of thalidomide in the treatment of discoid lupus erythematosus (DLE) and discusses four case studies that demonstrate its success. Two of the case studies included the addition of PLE. Patients who received PLE experienced a longer duration for complete clearance of symptoms; moreover, incorporating PLE helped to lower the thalidomide dosage and thus reduce its side effects.	[61]
Clinical study	PMLE	In this prospective study, a standardized extract of *P. leucotomos*, along with nicotinamide, vitamin D and zinc was orally administered to 15 patients suffering PMLE. These patients had not achieved symptom control through the use of only topical photoprotection. Administering a standardized extract of *P. leucotomos*, nicotinamide, vitamin D and zinc orally in conjunction with appropriate topical photoprotection offers a safe and effective alternative for preventing and minimizing the frequency and severity of outbreaks in individuals with PMLE.	[55]
Other photodermatoses: Chronic Actinic Dermatitis			
Case report	Chronic actinic dermatitis	In this study, a case of a patient with chronic actinic dermatitis (CAD) who showed only partial improvement with dupilumab is described. Initial management included sun avoidance and photoprotective therapy, which included topical Fernblock^®^, among others. The CAD did not improve, and the treatment continued with topical corticosteroids, immunomodulators, and systemic immunosuppressive agents. The continued implementation of photoprotection measures such as oral supplements, including oral and topical PLE, is recommended due to their proven efficacy as adjuvants to the above-mentioned pharmacological treatments.	[71]
Other studies with PL extracts in photodermatoses: non- Fernblock^®^ PL extracts			
Review	Rosacea	The purpose of this study is to explain the origin of rosacea, with a particular focus on the influence of UV radiation and exposome on the development of this skin condition. Additionally, this review highlights the importance of non-pharmacological approaches, with specific emphasis on photoprotection strategies in managing rosacea, using, for example, an extract of *P. leucotomos*.	[72]

#### 3.2.3. Pigmentary Disorders

The color of skin is determined by several pigments, one of which is the melanin produced by melanocytes, whose primary function is to protect from UVR. Pigmentary skin disorders are frequently encountered in the practice of adult medicine and include both hypopigmentation and hyperpigmentation alterations. Although most of these disorders are rarely associated with health risks or systemic diseases, they can sometimes lead to severe of life-threatening pathologies such as melanoma. Moreover, they can lead to negative effects on quality of life and become a source of discomfort and emotional stress for patients. Despite their frequency, these disorders remain challenging to treat [73].

Among these skin disorders, vitiligo and melasma are two of the most frequent. Both affect the skin’s appearance and can have notably adverse effects on an individual’s health-related quality of life. Melasma is in fact the skin pigmentary disorder with the highest incidence [74].

##### Vitiligo

Vitiligo is a common depigmenting skin disorder characterized by the selective loss of melanocytes. It has recently been reported that the combination of NB-UVB and oral administration of PLE accelerates repigmentation and increases total repigmented area. Also, PLE can improve the efficacy of photo(chemo)therapy for vitiligo by reducing negative side effects and improving tolerance. PLE does this by preventing sunburn and phototoxic reactions, as shown in both in vitro and in vivo studies involving human and animal models [75]. A recent review suggests that innovative drug delivery methods have the potential to enhance the delivery of topical medications by improving their localization within the epidermis and increasing their effectiveness, providing an interesting possibility for future improvements in PLE’s efficacy [76].

Moreover, a recent review discussed the role of NRF2-ARE (antioxidant response element) pathway in the pathogenesis of vitiligo, since this pathway is involved in cellular defense against oxidative stress. The review listed several agents known as NRF2 activators that included PLE and may represent a potential therapeutic strategy for this pigmentary disorder [77]. In this sense, recent studies have demonstrated the ability of PLE to modulate NRF2 pathway, which could potentially help explain the positive clinical effects of PLE treatment in patients with vitiligo [10] (Table 3). Although further research is required to validate this hypothesis, this rationale could prove valuable not only in the treatment of vitiligo but also in other photodermatoses [78].

##### Hyperpigmentation Disorders

Hyperpigmentation is a term used to describe a skin condition in which an excess of pigment production occurs, resulting in dark spots or areas of the skin that appear darker than the rest. Hyperpigmentation can be caused by exposure to environmental pollution, hormonal therapies, cosmetics, contraceptive pills, pregnancy, photosensitizing agents and genetic susceptibility [79]. Some common forms of hyperpigmentation include melasma, environmental lentigo and post-inflammatory hyperpigmentation [80]. In recent studies on PLE and its ability to prevent hyperpigmentation, primary emphasis has been on melasma and the various factors that contribute to increased pigmentation (Table 3). This section will delve into the discussion of these studies and the factors involved in promoting hyperpigmentation.

Pigmentation resulting from exposure to VIS is a significant concern and thus a prominent area of research. VIS has been demonstrated to have various biological impacts on the skin, including DNA damage caused by the generation of ROS, the activation of pro-inflammatory cytokines, exacerbation of photo-induced conditions, and the promotion of pigmentation in melano-competent individuals. Thus, Mohammad et al., (2019) conducted a clinical study to assess the effectiveness of oral PLE treatment in preventing the adverse effects induced by VIS. The study involved twenty-two participants with Fitzpatrick skin phototypes IV–VI. On day 0, the subjects were exposed to VIS radiation. Immediately, as well as 24 h and 7 days after radiation, clinical evaluations using the Investigator’s Global Assessment (IGA) scoring system and spectroscopic assessments were conducted. The participants were then given a 28-day supply of PLE (480 mg daily). All subjects experienced immediate pigment darkening, persistent pigment darkening, and delayed tanning both before and after taking PLE, but instrumental assessments showed a statistically significant decrease in pigment darkening and delayed tanning in the PLE group. Results of this research indicate that PLE has an impact on VIS-induced effects. Therefore, PLE could be utilized as a supplementary approach to conventional photoprotection methods in order to safeguard against the detrimental effects of VIS [81]. These clinical findings can be explained by in vitro studies that have explored the potential mechanisms by which PLE prevents pigmentation caused by VIS. These studies have specifically examined the role of VIS, particularly blue light, in activating pathways associated with melanogenesis. A recent in vitro study conducted by Portillo et al. (2021) examined the effectiveness of Fernblock^®^ in mitigating pigmentation induced by blue light emitted by digital devices. The study revealed that Fernblock^®^ acts through multiple mechanisms, including the modulation of the p38 melanogenic signaling pathway, inhibition of photooxidation of melanin precursors and reduction of opsin 3 expression. These findings underscore the potential of Fernblock^®^ as a protective agent against the detrimental effects of visible light, particularly blue light [82]. While multiple in vitro studies have also shown that blue light can activate mechanisms associated with melanogenesis [83,84], further research is necessary to gain a better understanding of how chronic exposure and the prevalence of electronic devices in modern life can potentially impact melanogenesis. The controversy surrounding this topic remains significant, highlighting the need for more studies to provide comprehensive information about the clinical implications of long-term exposure to blue light and its effects on melanin production, particularly in heavily pigmented individuals who are especially prone to skin hyperpigmentation [85,86,87,88].

Recent studies have also focused on investigating the role of pollution in melanin production. Observations have revealed that the prevalence of pigmentation is higher in animals residing in polluted areas, particularly in urban-industrial sites. As an increasing number of individuals are exposed to elevated levels of air pollution, there is a possibility that environmental pollutants can influence melanogenesis in human skin. Epidemiological studies have indicated that exposure to air pollutants associated with traffic, such as diesel exhaust particles, is correlated with an increased occurrence of clinical manifestations of hyperpigmentation [89]. Ahn et al. observed that RNA-sequencing data from melanocytes exposed to particulate matter (PM) revealed an increase in the expression of molecules associated with the unfolded protein response. Notably, the IRE1α signaling pathway consistently showed upregulation and was found to be involved in PM-induced melanogenesis [90]. Similarly, the already mentioned study conducted by Gallego-Rentero et al. (2022) in skin cancer prevention demonstrated that skin exposure to photopollution not only results in DNA damage and oxidative stress but also triggers the activation of the melanogenesis pathway. This study provided compelling evidence of significant upregulation of opsin-3 in cells treated with BaP and subsequently exposed to UVA. However, when cells were pre-treated with Fernblock^®^ before irradiation, the expression of opsin-3 remained similar to basal level. This study revealed the capacity of Fernblock^®^ to prevent the activation of melanogenesis through the modulation of opsin-3 expression, specifically in response to photopollution [29].

With respect to melasma, the considerable quantity of existing evidence of PLE’s efficacy in preventing and reducing this disorder has meant that no new specific studies have been conducted over the past few years. The most recent studies have been narrative reviews that emphasize the use of PLE as an effective oral treatment for this pathology based on the evaluation of various measures such as the MASI (Melasma Area and Severity Index), MI (Melasma Index), melasma area and pigmentary intensity, among others. These reviews encompass a wide range of research in this area, including a notable reference study conducted by Goh et al. (2018). The study specifically validated the safety and effectiveness of oral PLE treatment as an adjuvant in the management of melasma. It was found to be particularly effective when used in combination with topical hydroquinone and sunscreen [91]. However, it is important to note that other recent studies have been conducted in this field using different PL extracts than Fernblock^®^. This is the case of the study conducted by Piquero-Casals et al. (2020) that examined the clinical outcomes of a combination of trichloroacetic acid, phytic acid and ascorbic acid peel as well as oral antioxidant supplements (including a non-detailed extract from PL) and topical treatments, for individuals with difficult-to-treat melasma. The findings indicate that this approach could be an effective solution for managing this patient group [92]. Research in the field of new active ingredients with antioxidant and antimelanogenic properties is ongoing on a daily basis, with the goal of discovering novel compounds for use in the cosmetics industry. As evidence of this, a recent article compared and assessed the protective potential of various extracts from Spanish ferns, using the standardized PLE as a reference. The research concluded that all fern extracts exhibit antioxidant activity and have the potential to inhibit hyperpigmentation through their anti-tyrosinase activity. Moreover, it concluded that hydrophilic extracts are more potent and effective than lipophilic extracts [93].

Recent studies are also beginning to investigating other fields, including the effects of agents like pollution, photopollution and blue light on pigmentation [86,89]. There is growing interest in exploring the potential of PLE in offering protection against these factors as they are closely associated with the development of pigmentary disorders and photoaging. As a result, the focus has expanded to explore novel areas that can contribute to a deeper understanding of pigmentation-related mechanisms and the potential benefits of PLE in this context.

**Table 3:** This table provides a summary of the scientific articles published in the last five years in the field of pigmentary disorders, specifically including references to, and conclusions about, PLE treatment.

Pigmentary Disorders			
Design	Pathology/Focus	Summary/Outcome	Study Reference
Reviews and book chapters	Pigmentary disorders	Pigmentary disorders (melasma, vitiligo, periocular hyperpigmentation, pigmented contact dermatitis and lichen planus pigmentosus) are over-represented in women in most societies. Their mechanisms and future therapies, including PLE, are reviewed.	[94,95]
Review	Pathways and ingredients involved in pigmentary disorders	The text provides an overview of the role oxidative stress plays in melanogenesis, particularly in response to skin exposure to UVR and VIS. It also discusses various pathways involved in pigmentary disorders. Additionally, the text offers guidance on effective approaches to modulate melanogenesis, including the use of vitamins, PLE, niacinamide and other options, such as lightening agents, that can aid in the better management of pigmentary disorders.	[80,96,97,98,99,100]
Review	Visible light and hyperpigmentation	These reviews focus on the role of VIS in hyperpigmentation disorders (melasma and PIH) and analyze the direct and indirect effects of blue light emitted by digital devices reported on in vitro and in vivo studies. Recent advances in understanding the protective role of PLE against UVA and VIS have led to it being cited as a reference agent in preclinical and clinical studies.	[95,101]
Review	Management of hyperpigmentation: dermatological procedures	This review focuses on the medical and dermoaesthetic procedures for hyperpigmentation and on challenges (resistance, recurrence, adverse effects) in the management of pigmentary disorders such as melasma and PIH. PLE is included as a reference compound due to studies that suggest its beneficial activity in treating dyschromias.	[102,103,104]
Review	Management of hyperpigmentation: topical	The review presents alternative ways to manage hyperpigmentation, including PLE, glutathione and thiamidol. It also provides a table summarizing the scientific evidence supporting their effectiveness.	[105]
Review	Management of hyperpigmentation: oral	The texts provide a review of literature on oral treatments for hyperpigmentation, with a specific focus on examining the clinical evidence that supports the use of several oral treatments, including PLE and others.	[106,107]
Review	Management of PIH	These reviews describe the first-line treatments for epidermal PIH (based on topical or oral skin lightening agents) and the available adjunctive therapies for patients refractory to first-line treatment or for dermal PIH (peels, laser, etc.). They also analyze the use of sunscreens for the treatment of melasma and PIH. PLE is included as a systemic skin-lightening agent, among others. It is noted that PLE is the only ingredient listed that does not have any reported adverse effects.	[108,109,110]
Clinical study	Skin pigmentation induced by VIS + UVA light	This study evaluates the role of topical antioxidants in protecting against VIS + UVA-induced effects in skin phototypes I–VI. Topical antioxidants inhibit erythema in phototypes I–III and reduce pigmentation in phototypes IV–VI. PLE is used as a reference compound to compare its antioxidant properties with those of other compounds.	[111]
Melasma			
Reviews and book chapters	Management of melasma: focus on topical and oral treatments	These articles discuss various techniques for treating melasma and evaluate the effectiveness and safety of ingredients like hydroquinone and tranexamic acid, as well as delivery systems that improve the depigmentation activity of certain agents. Additionally, all these reviews place PLE among the most effective oral agents recommended for melasma.	[79,112,113,114,115,116,117,118,119] Sistematic review and metanalysis: [120,121,122] Book chapters: [123,124]
Review	Management of melasma: focus on dermatological procedures	The main objective of these reviews was to analyze the available evidence on the efficacy and safety of microneedling alone or in combination with topical agents in reducing pigmentation and improving the quality of life for adult patients with melasma. Oral PLE treatment was included as a therapeutic option for melasma, suggesting that combined therapies tend to produce better results compared to monotherapy.	[125,126,127]
Review	Melasma	The review aims to provide a comprehensive understanding of the role of oxidative stress in melasma and the potential therapeutic benefits of various antioxidants for individuals with this condition. Here, PLE is considered a principal antioxidant for treating melasma, and a summary of clinical studies is included in these documents.	[117,128]
Review	Melasma pathways	The manuscript provides a review of the processes and pathways responsible for skin pigmentation, specifically the changes in melanogenesis that lead to melasma and resulting hyperpigmentation. The paper also discusses current treatments and therapies, including those administered topically, orally and through phototherapy, with a particular focus on the effects of cosmetics. PLE is included as a plant-based oral treatment.	[129]
Clinical study	Pigmentation disorders	This study aims to assess the effectiveness of PLE in preventing VIS-induced effects in human skin. PLE treatment induces a significant decrease in persistent pigment darkening and delayed tanning and reduces expression of several damage markers. The study provides scientific evidence to position PLE as a treatment for pigmentation disorders.	[81]
Vitiligo			
Review	Vitiligo treatments	These reviews discuss the pathogenesis of vitiligo and focus on treatment options for the disease including standard drug treatments, phototherapy (NB-UVB and PUVA) and the effectiveness of antioxidant therapies. Moreover, in the majority of cases, antioxidant therapies on their own are not capable of producing significant clinical improvements, except perhaps in mild cases, and they must be used alongside standard drug treatments in order to achieve noticeable outcomes, such as PLE in concomitance with NB-UVB or PUVA.	[76,130,131,132,133,134,135,136,137,138] Focused on PUVA: [139,140,141]
Review	Safety in vitiligo treatments	These reviews concentrate on the potential harm related to the use of medicinal plants, including PLE, and offer a summary of adverse drug reactions (ADRs) that have been reported in national and global individual case safety report databases.	[142,143]
Review	The role of NRF2-ARE in vitiligo	This paper examines the role of NRF2 in vitiligo and reviews several agents known as NRF2 activators, including PLE. It suggests that PLE’s efficacy in the treatment of vitiligo is the results of its activation of the NRF2 pathway.	[77]
Clinical study	PLE in vitiligo	This study involved 44 patients with generalized vitiligo who received either combined treatment of NB-UVB phototherapy and oral PLE or NB-UVB phototherapy and placebo. The results showed that oral PLE combined with NB-UVB improved repigmentation and increased response rate as compared to NB-UVB alone.	[75]
Other studies with PL extracts in melasma: non- Fernblock^®^ PL extracts			
Case report	Melasma	The aim of this research was to examine the practical outcomes of a treatment plan that combined multiple interventions: one conducted at home and the other performed in a clinical setting. The protocol consisted of using a peel containing trichloroacetic acid, phytic acid and ascorbic acid, in combination with oral antioxidant supplements containing an extract from PL. Additionally, topical products were provided to individuals with persistent melasma. The findings of the study suggest that this treatment protocol could represent an effective approach for managing melasma.	[92]
Chemical assay with different PL extracts	Photoaging, hyperpigmentation	This study evaluates the antioxidant capacity of different hydrophilic and lipophilic fern extracts. All ferns present antioxidant activity and potential to inhibit hyperpigmentation (antityrosinase activity). This report concluded that hydrophilic extracts are more potent and effective.	[93]

#### 3.2.4. Extrinsic Aging

Over the years, researchers have studied how factors such as sun exposure, air pollution, hormonal changes, diet and psychological factors can affect the skin by causing hyperpigmentation, worsening photodermatosis, promoting wrinkles or leading to the development of cancerous lesions [144]. While pollution can exacerbate certain skin conditions like atopy or eczema, a correlation has been demonstrated between pollution and the premature appearance of aging signs [145]. In recent years, there has been a growing interest in evaluating the potential preventive effects of PLE against pollution and solar-radiation-induced photoaging, resulting in an increase in the number of studies in this area (Table 4). In an in vitro study, human keratinocytes were treated with PLE and subsequently exposed to UVB radiation or fine particulate pollutants (PM2.5). The results showed that PLE promoted the NRF2 pathway and its downstream targets, counteracting oxidative stress and thus inducing an increase in cell viability. Additionally, PLE was found to decrease IL6, IL8 and melanin production induced by UVB exposure. These findings suggest that PLE may protect against photooxidative stress and other environmental pollutants [10]. Also, chronic exposure to UVR from sun is well-known to contribute to photoaging, inducing alterations which include clinical and histological changes and increased inflammation and oxidative stress. With respect to photoaging and activation of the melanogenic signaling pathway by photopollution, Fernblock^®^ was found to prevent morphological changes in mitochondria when exposed to a combination of BaP and UVA. It also inhibits the overproduction of ROS generated by the exposure to photopollution in both melanocyte and keratinocyte cell lines. These findings suggest that Fernblock^®^ has the potential to mitigate oxidative stress-induced damage and mitochondrial injury resulting from the combined effect of these harmful agents [28].

Although not as well-researched as UVR, VIS (and more concretely its blue component) and IR radiation have also been shown to cause skin alterations that lead to photoaging. In this last 5 years, research in this area has been directed towards investigating PLE’s action mechanisms against VIS, with a particular focus on its protective effects against blue light (Table 4). Not only recently, but beginning in 2018, Zamarrón et al. performed an in vitro study to assess the protective potential of PLE against harmful effects induced by VIS and infrared A (IRA) radiation. PLE mitigates VIS/IRA-induced alterations in different ECM components (MMP-1, CTSK, fibrillins 1 and 2 and elastin) and prevents damage induced by both radiations on cell morphology and viability. All this places PLE as a potential preventive strategy against damage caused by exposure to VIS and IR light [146]. Related to these findings, in 2019 a pilot research was conducted to evaluate the potential effect of oral Fernblock^®^ on photodamage induced by IR and VIS in human volunteers. After oral administration of Fernblock^®^, the participants were exposed to the combination of IR/VIS, and the expression of MMP1 from skin biopsies was analyzed. The results showed that Fernblock^®^ significantly reduced the overexpression of MMP1 induced by IR/VIS, indicating its potential antiphotoaging effect [147]. In another study previously mentioned in the pigmentation disorders section, 22 participants were exposed to VIS and then observed for 7 days to establish a baseline response. They then received a 28-day supply of PLE (480 mg daily) after the month was up, and VIS was radiated on the other side of the participants’ backs. Immunohistochemistry results indicated that PLE also attenuates VIS-induced damages such as inflammation (reduction of COX-2), alteration on structural integrity (MMP-1,2,9, which are responsible for the initial degradation of collagen) and alterations in pigmentation (MART-1) [81]. Results of these two studies reflect PLE’s ability to mitigate photoaging-related ECM degradation associated with VIS/IR exposure.

Concerning blue light from the VIS spectrum, excessive exposure blue light from digital devices could also play a role in premature aging. In this sense, the results from the already-mentioned in vitro work performed by Portillo et al. (2021) demonstrated that a dose of blue light simulating our usual exposure to digital devices causes damage to cell morphology and viability, even inducing alterations to mitochondrial membrane potential. The study demonstrated that preventive treatment with PLE reversed these effects, mitigating oxidative stress and promoting reversal to mitochondrial membrane potential basal status. [89].

Another study carried out with a different extract of *P. leucotomos* evaluated the photoprotective properties of an oral food supplement containing: vitamins A, C, D3, E, selenium, lycopene, lutein, green tea, *P. leucotomos* and grape extracts. Oral intake of this supplement increases MED and ferric reducing antioxidant power (FRAP). In general, it improves antioxidant status of skin and exerts photoprotective effects against photoaging [148].

In conclusion, research on the role of PLE in extrinsic aging has experienced the most significant growth compared to its other clinical applications. In addition, numerous studies have been dedicated to investigating mechanisms by which PLE exerts its protective effects against novel agents involved in aging such as pollution or VIS/blue/IR radiation (Table 4).

**Table 4:** This table provides a summary of the scientific articles published in the last five years in the field of extrinsic aging, specifically including references to, and conclusions about, PLE treatment.

Extrinsic Aging			
Design	Pathology/Focus	Summary/Outcome	Study Reference
Review	Skin aging	The present study reviews the literature on the underlying causes and pathophysiological processes of skin aging, healthy skin aging, and basic protective antiaging approaches. PLE is regarded as a reference antioxidant due to its phenolic content and it can be used orally or topically to counteract skin aging due to its capacity to reduce the harmful effects of UVR and its photoprotective, antioxidant, anti-inflammatory and antiaging properties.	[149]
Review	Photoaging	These reviews evaluate the ability of sunscreens to protect against photoaging and analyze the ideal characteristics of sunscreen, taking into account the impact of VIS and IR on skin aging (apart from UVR). This document includes PLE as a reference compound for oral photoprotection and its role in the prevention of photoaging.	[7,150,151]
Review	Photoaging pathway	This article discusses the mechanisms of photoaging, specifically in human dermal fibroblasts. PLE is included for its role in preventing photoaging caused by VIS and IR radiation by decreasing MMP-1 and Cat K levels and preventing changes in the expression of fibrillin 1, fibrillin 2, and elastin.	[152]
Review	Ingredients and photoaging	These reviews provide a summary of the pathways involved in skin aging and explore various therapeutic approaches that utilize natural actives. The reviews cite PLE as one of the main natural actives, extensively researched and with robust scientific evidence supporting its use and its role as a reference product.	[153,154,155,156,157,158,159,160,161,162,163]
Clinical study	Sunburn/photoaging/skin cancer	This study compares the efficacy of two identical sunscreens, one containing PLE and the other not. The presence of PLE in the formulation provided a significantly greater reduction in skin damage triggered by solar radiation (reduction of erythema, pigmentation, DNA damage, collagen breakdown and immunosuppression).	[29]
Clinical study	Photoaging induced by IR and VIS	This pilot study evaluates the effect of oral PLE against the IR and VIS-induced photodamage. PLE attenuates IR/VIS-induced MMP1 overexpression. This study reinforces the anti-photoaging potential of PLE.	[147]
Clinical study	Photoaging induced by VIS	Twenty-two participants were exposed to VIS before taking PLE and then observed for 7 days to establish a baseline response. After 28 days of taking PLE, VIS was administered to the opposite side of the participant’s back. Instrumental assessments showed a statistically significant decrease in persistent pigment darkening and delayed tanning in patients after PLE administration.	[81]
In vitro study	Photoaging induced by blue light	This study assesses the capacity of PLE to reduce pigmentation induced by blue light from digital devices. PLE prevents cell death, alteration of mitochondrial morphology and phosphorylation of p38 triggered by blue light. PLE also prevents melanin photodegradation through regulation of opsin-3 in melanocytes.	[82]
In vitro study	Pollution and aging	This study evaluates the potential of PLE to protect against xenotoxic stress related to exposure to fine particulate pollutants. PLE can reduce pollution-induced stress through modulation of NRF2 pathway.	[10]
Other studies with PL extracts in photoaging: non- Fernblock^®^ PL extracts			
Clinical study	Photoaging	This study evaluates the photoprotective properties of an oral food supplement containing: vitamins A, C, D3, E, selenium, lycopene, lutein, green tea, *P. leucotomos* and grape extracts. Oral intake of this supplement increases MED and FRAP. In general, it improves antioxidant status of skin and exerts photoprotective effects.	[148]

## 4. Conclusions and Future Perspectives

Strong scientific evidence supports the photoprotective properties of Fernblock^®^ in the prevention, attenuation and even reversal of phototoxic effects caused by solar radiation in the skin. In recent years, the pivotal role of the NRF2 in skin health has garnered significant attention. Its involvement in processes related to photoprotection, cancer prevention, and mitigation of photoaging has led to the exploration of its pharmacological modulation as a novel research approach [164]. Of particular interest is thus the recent findings regarding Fernblock^®^’s ability to modulate NRF2 [10]. A recent study by Tabolacci et al. (2023) demonstrated that UVA radiation upsets the redox balance, resulting in a notable decrease in the concentration of GSH. This decrease in GSH is believed to be closely associated with the modulation of the NRF2 pathway [165]. Based on these findings, the suggested crucial role of Fernblock^®^ in modulating the endogenous antioxidant systems of the skin (including CAT, GSH and GSSR) [166] may be directly linked to the modulation of NRF2. This connection could potentially explain Fernblock^®^’s efficacy in protecting against damage induced by solar radiation and other harmful agents. Also, the NRF2 signaling pathway exerts a negative regulatory effect on pro-inflammatory cytokines, chemokine releasing factors, MMPs and other inflammatory mediators such as COX-2 and iNOS. These mediators, either directly or indirectly, impact the relevant NF-kB and MAPK pathways as well as other networks involved in inflammation [167]. Fernblock^®^’s NRF2-modulatory mechanism can also be observed through its ability to inhibit transcriptional activation of NF-kB [11]. This underlines the extract’s role in modulating the interaction between these signaling mechanisms. These findings not only suggest the prevention of inflammation and oxidative stress, but there is also evidence that directly associates the modulation of DNA damage response by NRF2 through MAPK signaling [168]. In addition to discovering the role of NRF2 in DNA repair associated with the MAPK mechanism, another recent study revealed that the antioxidant and DNA protective effects are achieved through the modulation of the PI3K-AKT-NRF2 pathway. As a result of this modulation, a reduction in UVB-induced CPD formation was observed, leading to an enhancement in the activity and efficiency of the nucleotide excision repair (NER) pathway involved in DNA repair in irradiated skin cells [169]. In this regard, strong data have demonstrated that there are numerous mechanisms that may be involved in the regulation of NER pathway, with NRF2 being one of the key players [170]. Considering all these evidences, we can hypothesize that the effect of Fernblock^®^ on NRF2 modulation may be closely linked to its ability to repair DNA, potentially establishing a direct relationship with the reduction of CPDs not only at the time of irradiation but also after exposure (dark-CPDs) [28]. Perhaps both mechanisms could provide an explanation for Fernblock^®^’s modulator role over markers such as H2AX or p53, facilitating promoting DNA repair. Thus, the observed functions can be directly linked to the abundant polyphenols content found in the Fernblock^®^ extract. This family of chemical compounds has substantial evidence supporting its ability to mitigate the impact of UV radiation on various aspects, including DNA repair, reduction of cellular antioxidant levels, modulation of antioxidant signal transduction pathways, control of immunological response and protection of the extracellular matrix [171]. Understanding the rationale behind these action mechanisms may also help elucidate the effect of Fernblock^®^ in conditions such as XP or AK, where deficient NER mechanisms exist.

Additionally, there is a growing interest in investigating the potential of NRF2 modulation in mitigating skin aging, both in relation to chronological aging and as a response to photodamage. The research on Fernblock^®^ thus appears particularly relevant as it increases cellular antioxidant defenses, promotes DNA damage repair, reduces inflammation and stimulates skin repair [172]. NRF2 has been also associated with cutaneous pigmentation disorders that arise from redox imbalances, particularly in vitiligo as well as chronological hair greying. The dysregulation of NRF2 signaling has been implicated in these conditions, suggesting that oxidative stress and altered redox processes contribute to the loss of pigmentation in the skin and hair and promotes collagen degeneration [173]. Understanding the role of NRF2 in these disorders can provide insights into potential therapeutic approaches aimed at restoring normal pigmentation and collagen production in the management of these conditions [174].

An increasing amount of evidence suggests that the NRF2 signaling pathway is also dysregulated in many types of cancer, leading to abnormal expression of NRF2-dependent genes. Additionally, inflammation plays a crucial role in diseases associated with oxidative stress, particularly in cancer [167]. Therefore, we strongly believe that further investigation into the role of Fernblock^®^ in the NRF2 pathway would be highly compelling, as well as assessing its impact on the development of AKs and its potential progression to skin cancer.

The primary prevention strategy for these skin alterations typically involves the use of specific UVA and UVB filters for photoprotection. It is obvious, however, that Fernblock^®^ can improve photoprotection: not only does it counteract oxidative stress caused by solar radiation and aging, but it has also shown efficacy in reducing inflammation, melanogenesis and overall cellular damage in cultured keratinocytes exposed to pollutant particles in experimental models [28]. Furthermore, immunoprotection emerges as an additional marker of protection. Consequently, incorporating compounds that can modulate the immune response becomes crucial in preventing abnormal reactions that patients with dermatoses may experience upon sunlight exposure. Conducting further clinical research on Fernblock^®^ in photodermatoses thus becomes essential to provide further evidence supporting the potential immunomodulatory effects of this standardized extract.

In summary, there are several potential areas for future exploration regarding the role of Fernblock^®^. One area of interest is further investigating the relationship between Fernblock^®^ and NRF2 and its impact on other mechanisms such as DNA repair and autophagy. Understanding how Fernblock^®^ influences these processes can provide valuable insights into its broader effects on cellular health and skin protection. Additionally, exploring the potential effect of Fernblock^®^ in new pathologies, such as photodermatoses or pigmentary disorders, could uncover novel therapeutic applications for this ingredient. Continued research in these areas will contribute to a deeper understanding of Fernblock^®^’s mechanisms and potential benefits in various skin-related conditions.
